# Identification of lung‐specific genes by meta‐analysis of multiple tissue RNA‐seq data

**DOI:** 10.1002/2211-5463.12089

**Published:** 2016-06-16

**Authors:** Min Xiong, Daniel P. Heruth, Li Qin Zhang, Shui Qing Ye

**Affiliations:** ^1^ Division of Experimental and Translational GeneticsDepartment of PediatricsThe Children's Mercy HospitalUniversity of Missouri Kansas City School of MedicineMOUSA; ^2^ Department of Biomedical and Health InformaticsUniversity of Missouri Kansas City School of MedicineMOUSA

**Keywords:** genome, lung‐associated disease, lung development genes, lung‐specific genes, RNA‐seq

## Abstract

Lung‐specific genes play critically important roles in lung development, lung physiology, and pathogenesis of lung‐associated diseases. We performed a meta‐analysis of multiple tissue RNA‐seq data to identify lung‐specific genes in order to better investigate their lung‐specific functions and pathological roles. We identified 83 lung‐specific genes consisting of 62 protein‐coding genes, five pseudogenes and 16 noncoding RNA genes. About 49.4% of lung‐specific genes were implicated in the pathogenesis of lung diseases and 21.7% were involved with lung development. The identification of genes with enriched expression in the lung will facilitate the elucidation of lung‐specific functions and their roles in disease pathogenesis.

**Abbreviations**
AGER: advanced glycosylation end product‐specific receptor
CLDN18: surfactant‐associated protein J
lincRNA: long intergenic noncoding RNA
misc RNA: miscellaneous other RNA
NKX2‐1: thyroid transcription factor 1
OMIM: online Mendelian inheritance in man
SCGB1A1: secretoglobin, family 1A, member 1
SCGB3A2: secretoglobin, family 3A, member 2
SFTPA1: surfactant protein A1
SFTPA2: surfactant protein A2
SFTPB: surfactant protein B
SFTPC: surfactant protein C
SFTPD: surfactant protein D
SLC34A2: solute carrier family 34 member 2
snoRNA: small nucleolar RNA
TBX4: T‐box protein 4

Genes with tissue‐specific expression play significant roles in the physiology of multicellular organisms and associate frequently with human diseases 1. The lung is a complex respiratory organ necessary for the gas exchange of oxygen and carbon dioxide in mammals. It is the first line of defense against many pathogens and inhaled xenobiotics. Lung‐specific genes are involved in lung development, function, and lung disease pathophysiology 2, 3. Lung development, especially early stage, has been demonstrated to affect lung function and susceptibility to respiratory disease in later life 4. Thus, identification of genes expressed exclusively in the lung can provide insight into key physiological and pathological processes.

Previous microarray analyses have identified lung‐specific genes associated with both human and mouse lung development and disease pathogenesis 2, 3, 5. Analysis of existing microarray data from the Gene Expression Omnibus (GEO) public repository identified 11 lung‐specific genes across six human and mouse adult tissues 5. Expression profiling of 26 different tissues in 57 isogenic strains determined by the Affymetrix Mouse Genome 430 2.0 array identified 16 genes specific to the lung 2. Furthermore, genome‐wide microarray expression profiling of 38 normal human lung tissues ranging from 53 to 154 days post conception defined 3223 genes associated with lung development 3.

With the advent of next‐generation sequencing (NGS), RNA sequencing (RNA‐seq) has been used for the identification of both housekeeping and tissue‐specific genes 6, 7, 8. NGS is free from the limits of microarray technology, such as the bias due to probe selection, cross‐hybridization background, and signal saturation‐induced detection dynamic range limitation 9. The Human Protein Atlas integrated RNA‐seq transcriptomics and antibody‐based proteomics profiling to identify 190 elevated genes in the lung compared with their expression profile in other tissues 8, 10. Projects such as the genotype‐tissue expression (GTEx), BodyMap, functional annotation of the mammalian genome (FANTOM), and Human Protein Atlas provide thousands of multiple tissue RNA‐seq data for human, mouse, and rat 7, 8, 11, 12. However, due to the use of different sequencing platforms, as well as the species and number of tissue samples analyzed, it is hard to identify reliably every tissue‐specific gene. To overcome these problems, the Expression Atlas (https://www.ebi.ac.uk/gxa/home) remits RNA‐seq data into gene expression profiles across tissues 13. The aim of this study was to perform a meta‐analysis of multiple tissue RNA‐seq data obtained from the Expression Atlas to identify new and novel genes with enriched lung expression to facilitate the investigation of lung‐specific functions and disease pathogenesis.

## Materials and methods

### Data preprocessing

The gene expression profiles of 53 human GTEx tissues, 16 human BodyMap tissues, 56 human FANTOM tissues, 32 human Protein Atlas tissues, 64 mouse FANTOM tissues, and 10 rat BodyMap tissues were downloaded from the Expression Atlas (https://www.ebi.ac.uk/gxa/home) 13. The Expression Atlas from the European Bioinformatics Institute adheres strictly to the policy that collection and dissemination of human genome data are consistent with the informed consent of the participants of the study and have been granted ethical approval by the appropriate institutional ethics committees. The Expression Atlas utilized iRAP for RNA‐seq analysis to integrate existing tools for filtering, mapping reads, and quantifying expression. Quantile normalization was used to make distributions of expressions equalized in each biological replicate and then average gene expression levels across biological replicates. These normalization expression data were then collected as initial data.

### Shannon entropy for determining lung‐specific genes

Shannon entropy (*H*) for each gene was calculated in the preprocessed tissue expression data according to the method of Schug *et al*. 14. At first, we defined the relative expression of each gene *P*_*ij*_ in *N* tissues:

$$P_{ij}=\frac{E_{ij}}{\sum_{1\leq j\leq N}E_{ij}}$$

where *E*_*ij*_ is the expression of gene *i* in tissue *j*. Then, Shannon entropy *H*_g_ was computed for the entropy of gene's expression distribution:

$$H_{g}=\sum_{1\leq j\leq N}-P_{ij}\log_{2}P_{\mathrm{ij}}$$

To identify tissue‐specific genes, we defined those genes with *H*_g_ < 2 as tissue‐specific genes. Then, we classified tissue‐specific genes with the highest *E*_*ij*_ in lung as lung‐specific genes.

### Homology analysis

Human, mouse, and rat orthology information was retrieved from Ensembl by BioMarts (http://www.ensembl.org/index.html) 15. The gene orthology predictions were generated by a pipeline, where maximum likelihood phylogenetic gene trees play a central role.

### Gene function analysis

To identify biological processes and potential pathological properties of lung‐specific genes, we applied Database for Annotation, Visualization and Integrated Discovery (DAVID) (https://david.ncifcrf.gov/) 16 and ingenuity pathway analysis system (IPA; Ingenuity Systems, Inc., Redwood City, CA, USA) to perform gene ontology, OMIM, genetics‐associated analyses and network enrichment. The transcription factor prediction database (DBD) 17 and the database of essential genes (DEG) 18 were employed to annotate transcription factors and essential genes.

### Automated literature search

PubMatrix analysis (http://pubmatrix.grc.nia.nih.gov/) 19, a multiplex literature mining tool, was used as described previously 20 to build the relationship between our gene list with lung function and lung‐associated diseases in PubMed.

## Results and Discussion

**Figure 1:**
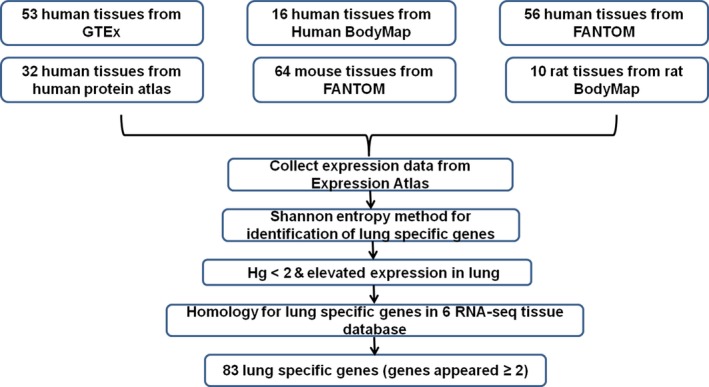
Workflow for the identification of lung‐specific genes.

**Figure 2:**
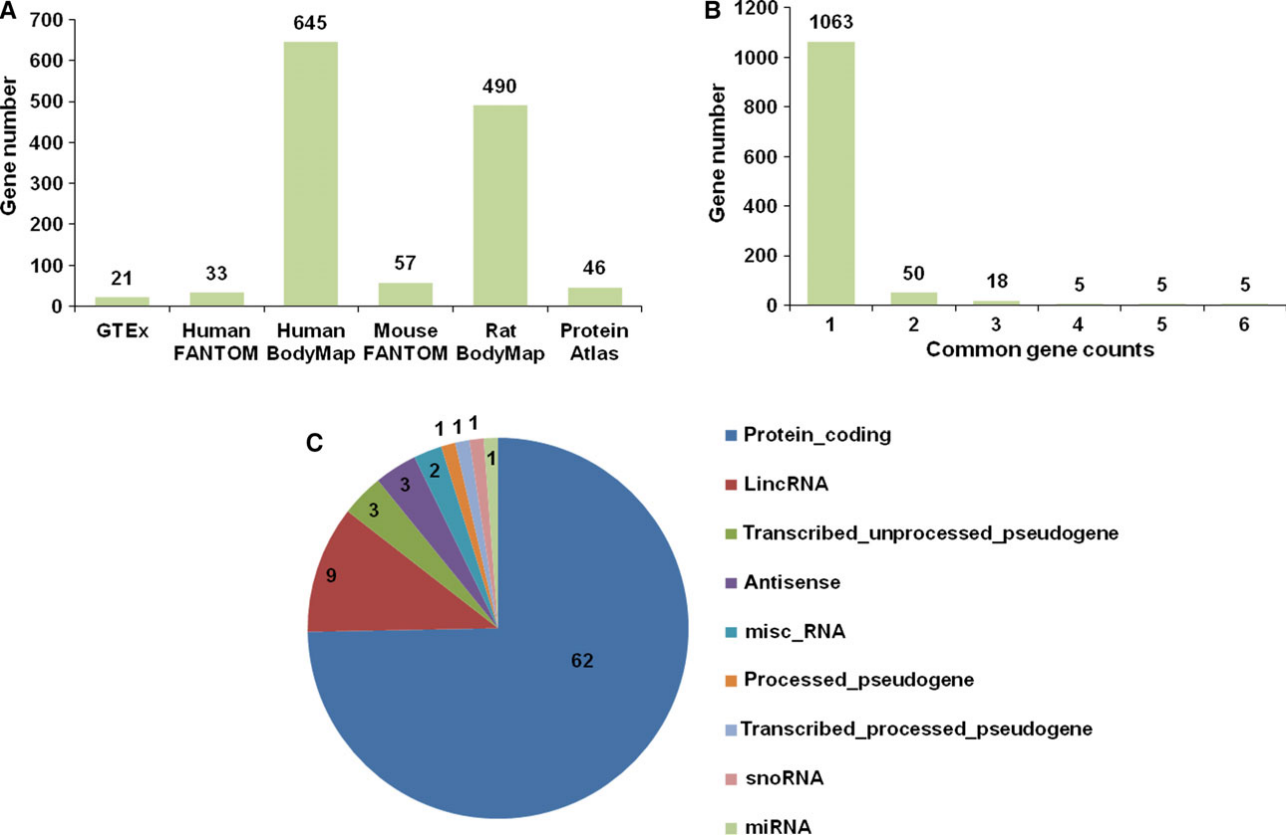
Discovery of lung‐specific expression genes. (A) Lung‐specific gene number identified in each of six data sets. (B) Number of genes common between one and six data sets. (C) Biotype of the 83 lung‐specific genes as defined by appearing in at least two data sets.

We performed a meta‐analysis of six RNA‐seq data sets of human, mouse, and rat tissues compiled by the Expression Atlas to identify lung‐specific genes by (a) Shannon entropy (*H*_g_ < 2), (b) elevated expression in lung compared with other tissues, and (c) detection of a gene in at least two data sets (Fig. 1). We found 21 lung‐specific genes in the human GTEx data set, 33 in human FANTOM, 645 in human BodyMap, 57 in mouse FANTOM, 490 in rat BodyMap, and 46 in Human Protein Atlas (Fig. 2A). The majority of these genes were expressed in only one database (Fig. 2B & Table S1). To increase stringency, we required that a lung‐specific gene must be expressed and listed in two or more databases. Using these criteria, we defined 83 lung‐specific genes (Table S2). The SFTP gene family, which encodes lung surfactant proteins, was represented by expression of five genes (*SFTPA1*,* SFTPA2*,* SFTPC*,* SFTPB*, and *SFTPD*) in at least five databases. These genes play essential roles in surfactant homeostasis, lung development, and in the defense against respiratory pathogens 21, 22, 23, 24. *SFTPA1*,* SFTPC*, and *SFTPD* were also detected previously as mouse lung‐specific genes 2. Thus, the detection of the SFTP gene family serves an internal validation control for our study. Figure 2C shows that 62 of the genes identified in our study are protein‐coding genes. A DEG database search of these genes revealed that nine of the protein‐coding genes are essential genes, including the *TBX4* and *NKX2‐1* transcription factors (Table S2).

Microarray expression analysis of human and mouse tissue by Song *et al*. 5 identified six lung‐specific genes (*SFTPC*,* SFTPB*,* SCGB1A1*,* AGER*,* SLC34A2*, and *CLDN18*) that were also identified in our study. In addition, 32 of the 62 lung‐specific protein‐coding genes (51.6%) detected in our study correspond to genes with elevated expression in lung tissue identified by the Human Protein Atlas transcriptomics and proteomics profiling study 8, 10 (Table S3). Further analysis of the Human Protein Atlas study revealed that 17 of 20 lung tissue‐enriched genes, six of 117 lung tissue‐enhanced genes and nine of 53 lung group‐enriched genes overlapped with our lung‐specific gene list. These results support our further approach as a powerful method for the identification of tissue‐specific genes.

**Figure 3:**
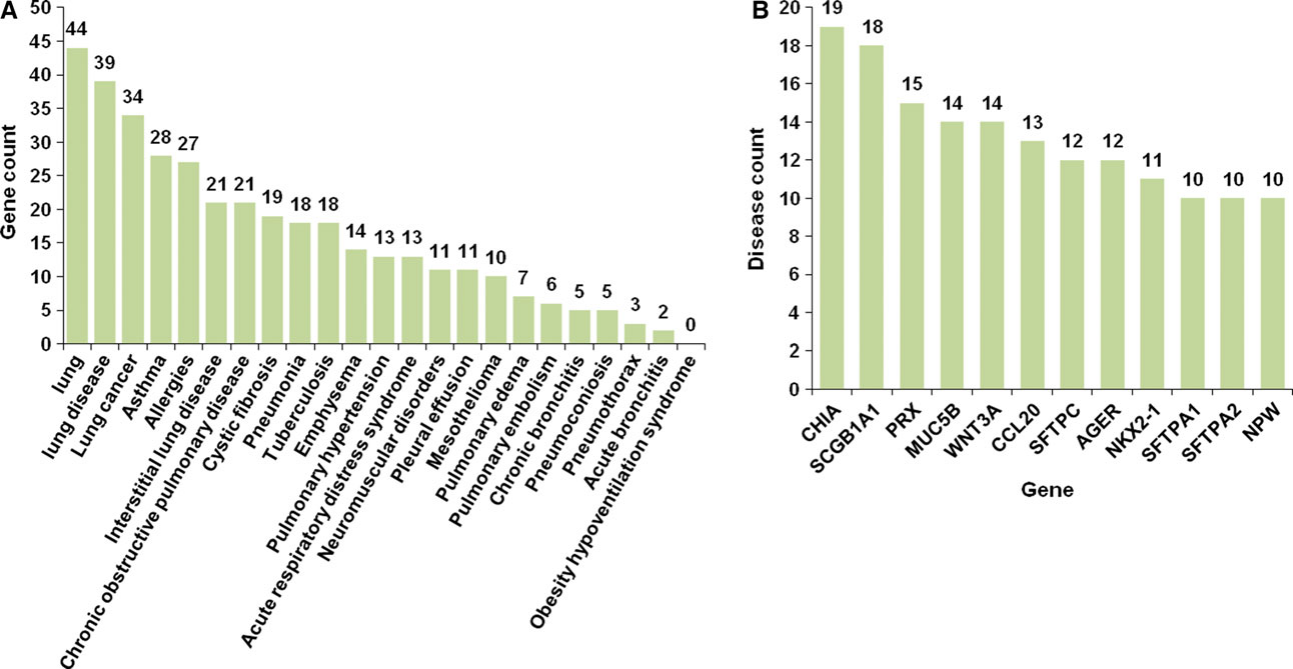
Lung‐specific disease genes. (A) Gene count linked to lung, lung disease and 21 lung‐associated diseases identified by PubMatrix analysis; (B) Lung‐associated disease count of the top 12 lung‐specific genes identified by PubMatrix analysis.

To identify the relevance of our lung‐specific genes to lung physiology and associated diseases, we linked our 83 lung‐specific genes to the terms ‘lung’, ‘lung disease’, and 21 distinct known lung diseases using the PubMatrix tool 19. This approach identified 45 genes as being previously linked to the terms ‘lung’ or ‘lung disease’. Forty‐four lung‐specific genes (53.0%) as previously linked to lung genes (at least one citation with the term ‘lung’), which justifies further the suitability of meta‐analysis of multiple tissue RNA‐seq data to identify lung‐specific genes (Table S4 & Fig. 3A). Thirty‐nine lung‐specific genes (47.0%) linked to ‘lung disease’ and 41 lung‐specific genes (49.4%) linked to at least one of 21 known lung diseases, further demonstrating that lung‐specific genes are associated with lung disease pathologies (Fig. 3A). Analysis of the 21 lung disease categories reveals that 34 genes linked to lung cancer, 28 genes linked to asthma, and 27 genes linked to allergies. Twelve lung‐specific genes were shared by at least 10 lung diseases (Fig. 3B). Lung‐specific protein TSA1902 (*CHIA*) contributes to inflammation in response to IL‐13, stimulates chemokine production by pulmonary epithelial cells and protects lung epithelial cells against apoptosis 25, 26. *CHIA* linked to 19 lung diseases; it has not yet been associated with emphysema and obesity hypoventilation syndrome. Secretoglobin, Family 1A, Member 1 (*SCGB1A1*) encodes a member of the secretoglobin family of small secreted proteins. It is found predominantly in the respiratory bronchioles 27. *SCGB1A1* has been implicated in anti‐inflammation 28, which linked to 18 lung diseases in our study.

Our study has also identified 38 lung‐specific genes with no previous PubMatrix literature links to the terms ‘lung’ or ‘lung disease.’ The list of novel lung‐specific genes consisted of 18 protein‐coding genes, five pseudogenes, and 15 noncoding RNA (ncRNA). The function of these protein‐coding genes involved with fatty acid metabolic process, apoptosis regulation, and cell adhesion (Table S5). While protein‐coding genes have been well studied in relationship with cellular function and disease pathology, the roles of pseudogenes and ncRNA in gene regulation and disease pathogenesis are just now starting to be elucidated. The identification of 38 potentially novel lung‐specific genes provides new opportunities to investigate lung physiology and disease.

ncRNA play important roles in lung development, gene expression, and translation regulation. Dysregulation of ncRNA is associated with lung dysfunction 29, 30. In our study, 16 lung‐specific ncRNA (9 lincRNA, 2 misc RNA, 3 antisense RNA, 1 microRNA, and 1 snoRNA; Fig. 2C & Table S2) were identified. However, most of the lung‐specific ncRNA genes remain poorly defined.

**Figure 4:**
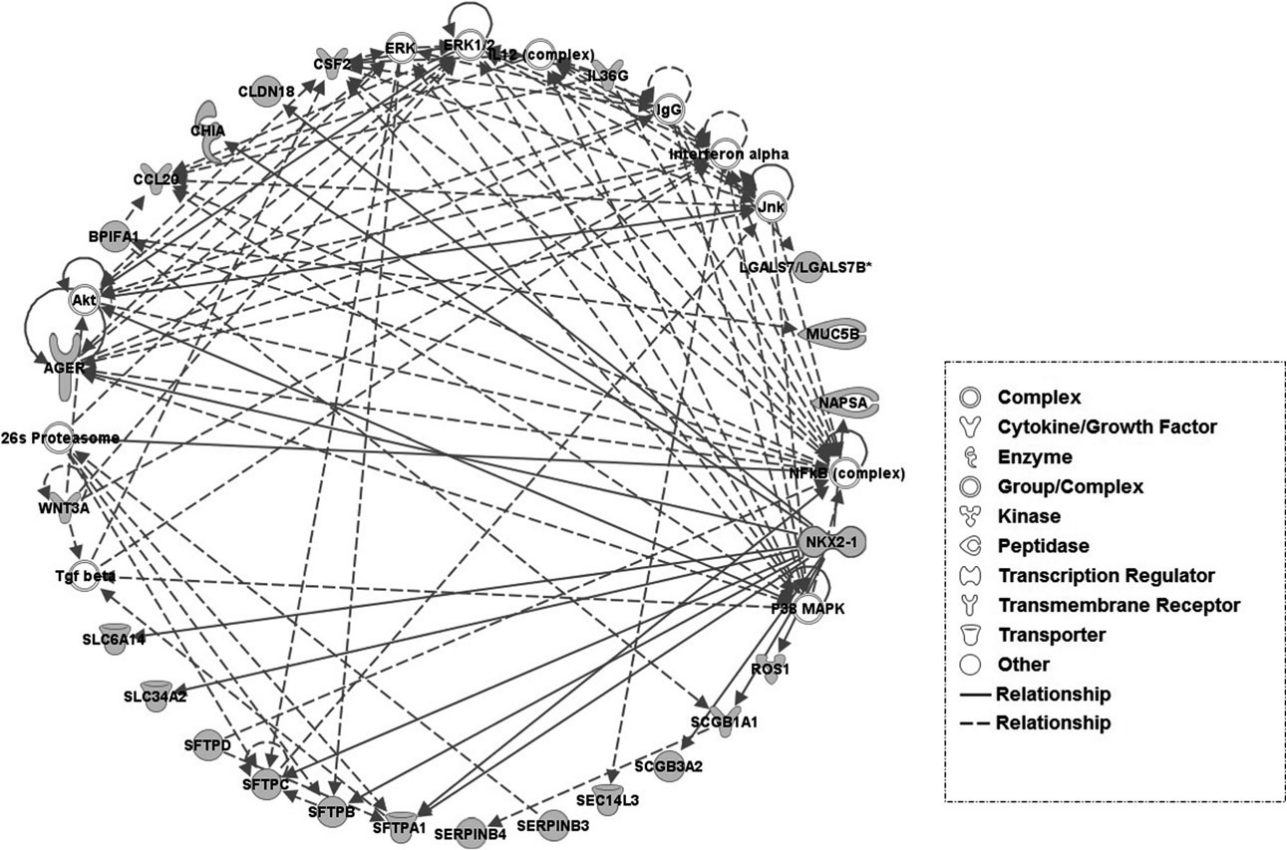
Network of respiratory disease, cell morphology, embryonic development. Gray node = lung‐specific genes; white node = other genes; solid line = direct interaction; dot line = indirect interaction.

We next analyzed the 83 lung‐specific genes through IPA. Twenty‐four lung‐specific genes are associated with the ‘respiratory disease, cell morphology, embryonic development’ network (Fig. 4). Of note, transcription regulator NKX2‐1 plays a role in lung development and surfactant homeostasis 31, 32. In the network, NKX2‐1 regulates 12 lung‐specific genes’ expression directly. The 83 lung‐specific genes identified in present study have been annotated in detail in Table S5. Interestingly, biological process enrichment showed that the lung‐specific genes identified in this study play an important function in respiratory gas exchange, immune response, tube development, and lung development (*P* value < 0.05; Table S6). These results suggested that our lung‐specific genes support lung function. OMIM disease analysis revealed that mutations within six genes (*SLC34A2*,* SCGB1A1*,* SCGB3A2*,* SFTPB*,* SFTBC*, and *SFTPA1*) cause pulmonary‐associated diseases (Table S7). Genetic database enrichment also showed that the lung‐specific genes identified in this study are involved with lung‐associated diseases (e.g. bronchopulmonary dysplasia, pulmonary fibrosis and respiratory distress syndrome, and asthma; Table S8), which also support that lung‐specific genes play important roles in lung‐specific functions and disease pathogenesis.

**Figure 5:**
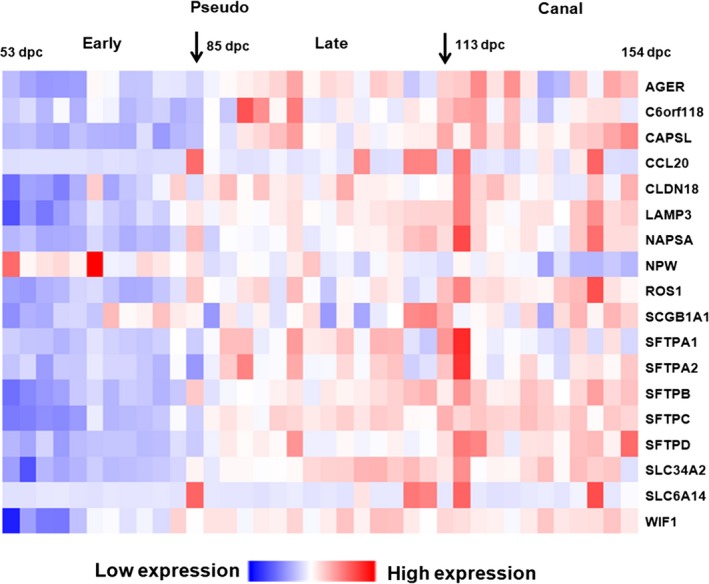
Gene expression during human lung development. Expression profiles for 38 human fetal lung tissues were extracted from GEO: GSE14334. The expressions of 18 lung‐specific genes identified in this study are mapped against lung development. Sample order followed development time, which increases from left to right. Arrows represent two developmental time points of 85 and 113 days post conception (dpc).

Kho *et al*. 3 defined 3223 genes as lung development genes by transcriptional profiling of 38 human normal lung tissues ranging from 53 to 154 days post conception. Eighteen genes identified in our study corresponded to 31 probes from the Kho study. The expression pattern for most of the 18 genes increased from the early to late pseudoglandular stages of lung development (Fig. 5). The subset of 18 genes, includes five lung surfactant protein genes (*SFTPA1*,* SFTPA2*,* SFTPC*,* SFTPB*, and *SFTPD*) supporting further the importance of surfactants in lung development. Sixteen of the 18 genes linked to ‘lung disease’ genes by PubMatrix analysis, demonstrating the association of lung development genes in disease pathogenesis 4.

Meta‐analysis of RNA‐seq data is a powerful tool for the detection of tissue‐specific genes; however, limitations exist in our study. The RNA‐seq data was obtained from different species, different tissues samples, and different tissue sample numbers, which can complicate the analysis. In our results, fewer lung‐specific genes were identified in the data sets with a larger number of tissues analyzed, indicating that the analysis of fewer tissues may overestimate the number of lung‐specific genes. In addition, analysis of developmental genes was performed on a single data set ranging from 53 to 154 days post conception. Thus, analysis of additional studies with increased time points will strengthen the identification of genes involved in lung development.

## Conclusions

In this study, we used a meta‐analysis of multiple tissue RNA‐seq data to identify 83 genes with enriched lung‐specific expression profiles, including 62 protein encoding genes, five pseudogenes, and 16 ncRNA genes; most of which have not been previously reported as lung‐specific transcripts. We expect that further studies of these newly identified lung‐specific genes, especially the ncRNA, will lead to new biomarkers for lung development and disease.

## Author contributions

MX and DPH performed Meta‐analysis and drafted the manuscript. SQY and LQZ conceived the study and critically revised the manuscript.

## Supporting information


**Table S1.** One thousand one hundred and forty‐six lung‐specifc genes of six data sets.

**Table S2.** Eighty‐three lung‐specific genes.

**Table S3.** Thirty‐two lung‐specific genes confirmed by 190 lung‐elevated genes of Human Protein Atlas.

**Table S4.** The relationships between lung‐associated diseases and 83 lung‐specific genes identified by PubMatrix analysis.

**Table S5.** Function annotation table of 83 lung‐specific genes.

**Table S6.** GO biological processes enrichment of 83 lung‐specific genes.

**Table S7.** OMIM disease information of 83 lung‐specific genes.

**Table S8.** Genetic‐associated diseases enrichment of 83 lung‐specific genes.
